# Oral and oropharyngeal high-risk HPV prevalence, HIV status, and risk behaviours in a cohort of South African men who have sex with men

**DOI:** 10.3934/publichealth.2022010

**Published:** 2021-12-03

**Authors:** Harshita B Mistry, Ramokone L Lebelo, Fulufhelo Matshonyonge, Maphoshane Nchabeleng, Matsontso Mathebula, John-Paul Bogers, Neil H Wood

**Affiliations:** 1 Department of Periodontology and Oral Medicine, School of Oral Health Sciences, Sefako Makgatho Health Sciences University, Pretoria, South Africa; 2 HIV and Hepatitis Research Unit, National Health Laboratory service, Department of Virology, Sefako Makgatho Health Sciences University, Pretoria, South Africa; 3 Department of Microbiology, Sefako Makgatho Health Sciences University, Pretoria, South Africa; 4 Mecru Clinical Research Unit, Sefako Makgatho Health Sciences University, Pretoria, South Africa; 5 Faculty of Medicine and Health Sciences, Applied Molecular Biology Research Group (AMBIOR), Laboratory of Cell Biology and Histology, University of Antwerp, Belgium

**Keywords:** MSM, oral human papillomavirus (HPV), oropharyngeal HPV, human immunodeficiency virus (HIV), oral sex, rimming

## Abstract

Data lag is evident when observing studies focussing on human papillomavirus (HPV) prevalence in the head and neck of men who have sex with men (MSM) in Southern Africa. Sexual behaviours other than anal intercourse, and associated factors are similarly underreported. HPV vaccination has not yet commenced for this population group. One hundred and ninety-nine MSM were enrolled in this study. Participants completed a questionnaire followed by a clinical oral examination, and a rinse-and-gargle specimen in Thinprep^®^ vials containing Preservcyt^®^ solution was collected. Detection and genotyping for high-risk HPV were done by an automated system (Abbott^®^ m2000sp). Six percent of MSM in this cohort had high-risk HPV present in the mouth/oropharynx. This cohort averages 29 years of age, more than half were unemployed (53.3%), and 66.8% were human immunodeficiency virus (HIV) seropositive. The most common sexual practice was anal sex (69.4%) followed by oral sex (28.6%), and by rimming (9.6%). A significant association between oral insertive sex and oral/oropharyngeal HPV status was demonstrated (p = 0.0038; phi coefficient = 0.20). An incidental but significant association between rimming and HIV status was found (p = 0.0046; phi coefficient = 0.19), and HIV seropositive participants had higher oral/oropharyngeal HPV presence. The HPV prevalence of 6% reported in this study is in alignment with global reports. The prevalence of oral/oropharyngeal HPV in this MSM cohort was influenced by sexual practices. MSM participants who practiced rimming appear to be at higher risk of HIV acquisition. Given the transmission routes of HPV in this vulnerable population, vaccination must be urgently studied as an intervention for prevention.

## 1. Introduction

The Human papillomavirus (HPV), a non-enveloped DNA virus, is the most common sexually transmitted agent. The virus is responsible for a variety of benign lesions, potentially malignant lesions, and malignancies in the form of squamous cell carcinoma (SCC) of different anatomic sites lined by mucosa [1]–[3]. The association with SCC of the head and neck anatomic region, specifically the oropharynx, is well established [4].

Studies on head and neck HPV infection involving MSM populations are very limited. The 2019 *Information Centre on HPV and Cancer (ICO) Report* [5] demonstrates that there are no reliable data for HPV infection of the head and neck anatomical region in healthy South Africans. Only two studies from South Africa relating to head and neck SCC were included in the report. Only one study from Cape Town investigated oral/oropharyngeal HPV presence in MSM, along with other anatomic sites [6]. The data lack underpins the urgent need for baseline studies in the Southern African region. This plays a role in increased heterogeneity making the syntheses of meta-analyses for systematic reviews for this geographic area particularly challenging.

MSM are a high-risk vulnerable population and are more prone to acquiring oral/oropharyngeal HPV infection due to the practice of high-risk sexual behaviours. These individuals are also at a higher risk to acquire HIV infection. This study investigated the presence of oral/oropharyngeal HPV in a group of 199 MSM and forms an important baseline contribution to the Southern-African database.

## 2. Materials and methods

This cross-sectional descriptive study was performed in the Clinical Research Unit (MeCRU) and Department of Virology at Sefako Makgatho Health Sciences University in Pretoria, South Africa. This unit falls within a 28 km radius of four townships (Ga-Rankuwa, Soshanguve, Mabopane and Mothutlung). The villages of Winderveldt and Mmakau, and the North-Western suburbs of Pretoria form part of the catchment area served by the unit.

### 2.1. Inclusion of participants

Men who self-declared to have sex with other men, who were 18 years or older, resided in targeted areas, and who consented to participate in this study were included. A recruitment team comprising of a community liaison officer (CLO) and 2 other recruiters was formed. During the initial phase of the study they established links with community based organisations to discuss and educate communities about the realities that needs to be faced concerning the MSM population. Local health care facilities were consulted to reinforce the links that the recruitment team had already established. Consultations with other men's health organizations, enabled training of the health care workers at the facilities to educate and equip them, to create a MSM-safe and friendly environment at the health care facility. Contact with the MSM occurred in environments where they felt comfortable to interact with staff/data collection teams.

### 2.2. Data and specimen collection

Participants received an information leaflet upon arrival at the research unit. Thereafter a group discussion took place and informed consent was obtained. Participants completed a self-administered questionnaire. Demographic data were collected followed by intra-oral examination and rinse specimen collection. Current sex practice was defined as any of the described forms of sexual contact having occurred in the 6 months leading up to participant enrolment. The examination was performed in a fluorescent lighted room with a LED headlamp using standard infection control procedures. Patients were referred to the dental hospital for diagnosis and management of any abnormalities seen during their examination.

After the clinical oral examination, participants rinsed their mouth with saline for 15 seconds and gargled for another 15 seconds, then spit the contents into a Thinprep^®^ vial containing the Preservcyt^®^ solution. Rinse specimens were then delivered to the laboratory on the same day for processing which was uneventful. The specimens were processed according to standard operating procedures of the Department of Virology, Health Science Laboratory, Sefako Makagatho Health Sciences University.

### 2.3. HPV-testing

High-risk (HR)-HPV detection and genotyping was done using the Abbott® m2000TM RealTime system which is a qualitative assay used to detect 14 HR-HPV types (16, 18, 31, 33, 35, 39, 45, 51, 52, 56, 58, 59, 66, 68), and to genotype HPV 16 and HPV 18. This real-time, multiplex PCR detection kit was designed to provide clinically meaningful results through the multiplex PCR, and through the clinically-based assay cut off. The assay is clinically optimised for cervical cancer screening and also optimized for high reliability through the probe specificity design and Human-Beta Globin internal control. The clinically-based assay cut-off is utilized in this study for the detection of HR-HPV in oral washes.

### 2.4. Data collection and management

It was calculated that at least 123 MSM should be recruited for an expected frequency of 25% MSM in the population and taking into account globally and locally reported prevalence rates for oral/oropharyngeal HPV (5% precision, 95% confidence level). A sample size of 200 participants was finally decided on, but one participant was deemed a screen failure. The screen failure was defined as an enrolled participant that subsequently withdrew consent for sample collection.

The data were extracted from the questionnaire to protect the data integrity and anonymity and entered into a Microsoft^®^ Excel^®^ database. Laboratory results were recorded on a data collection sheet and included data on the detection of the 14 HR-HPV types. Data from the intra-oral examinations were noted in each participant's file and entered into the spreadsheet.

Descriptive data were summarized as categorical values by frequency and percentage tabulation. Continuous variables were summarized by the mean, standard deviation where applicable and median. The chi-squared test was used to assess the relationship between HPV status and race, HIV status, current sex practices, number of partners and Socio-economic status. The relationship between HPV status and age was assessed by the Wilcoxon rank sum test, since the data did not meet the assumption of the independent samples t-test. The strength of associations was measured by the Cohen's d for parametric tests and the r-value for the non-parametric tests and reported where applicable. Data analysis was carried out using SAS^®^ (version 9.4 for Windows^®^) and the 5% significance level was used.

### 2.5. Ethical approval

All procedures performed in studies involving human participants were in accordance with the ethical standards of the institutional and/or national research committee and with the 1964 Helsinki declaration and its later amendments or comparable ethical standards. Consent was informed and obtained in writing from all individual participants included in this study.

This project was specifically approved by the Sefako Makgatho Health Sciences University Research Ethics Committee, clearance number SMUREC/D/300/2016.

## 3. Results

Twelve participants (6%) had detectable oral/oropharyngeal HR-HPV, with 1 of these being HR-HPV-16. HIV serological tests revealed that 66.8% of the participants were infected with HIV and of these 10 (7.5%) were HPV positive compared to only 2 (3.1%) of those who were HIV negative. The majority (105) of the participants were unemployed (53.3%) compared to 36.0% who were employed and there was a number of students enrolled in the study (10.7%). The mean age of participants was 29 years and the median age 27 years (IQR 24–31y; range 18–47y). The majority of participants where younger than 35 years of age and there was a decline in the age demographic just after 27 years of age. Most of the participants self-identified as black (98.5%) and the remainder as dual heritage (1.5%). Of those who identified themselves as black 6.2% were HPV positive while none of the dual heritage individuals had HPV infection (Table 1). One participant was a screen failure which meant that even though the patient agreed to participate and complete some aspects of data collection, they withdrew reducing the participant number from 200 to 199.

**Table 1.** Comparison of HPV status according to demographic data.

		Overall		HPV status				p-value
				Negative		Positive		
Variable	Category	N = 199	%	N = 186	row (%)	N = 12	row (%)	
Race*	Black	196	98.5	183	93.8	12	6.2	>0.99
	Dual heritage	3	1.5	3	100.0	0	0.0	
Social Economic Status (SES)	Unemployed	105	53.3	99	94.3	6	5.7	0.41
	Employed	71	36.0	65	91.5	6	8.5	
	Student	21	10.7	20	100.0	0	0.0	
	Unknown	2						
HIV status	Positive	133	66.8	123	92.5	10	7.5	0.34
	Negative	66	33.2	63	96.9	2	3.1	

Note: * Self-declared by participants.

The mean age in years of the oral HPV-positive participants was 30.3; the standard deviation was 6.2. The median age for oral HPV positive participants was 29. The age range between the oral HPV positive and negative was the same; positive being between 20–42 years and negative being between 18–47 years (Table 2).

**Table 2.** Comparison of age and HPV status amongst MSM.

Age (y)										p-value
HPV	N Obs	N	Mean	Std Dev	Median	IQR		Min	Max	
Negative	186	186	27.9	6.4	27	24	31	18	47	0.15
Positive	12	12	30.3	6.2	29	27	34	20	42	

The most commonly reported ‘current sex practice’ was anal sex (69.4%) followed by oral sex (28.6%), and by rimming (9.6%) (Table 3). Fourteen percent of participants who practised oral sex were positive for HR-HPV, which was statistically significant (p = 0.0057). About 67.5% of the participants reported having multiple partners in the 6 months preceding the study. Almost half (47.1%) of the participants reported to have had 3 or more sexual partners in the 6 months before enrolment. Only 4 out the 90 participants that had 3 or more partners during these 6 months had detectable oral/oropharyngeal HPV. Nine participants reported to have no partners in the past 6 months before enrolling into this study, and 2 of these were positive for oral/oropharyngeal HPV.

Many participants (80.9%) have practiced oral receptive sex (participant's mouth), 60.8% have performed oral insertive sex (partner's mouth), while 52.8% have performed both. There has been a significant association between oral insertive sex and oral/oropharyngeal HPV status (p = 0.0038; phi coefficient = 0.20). Of those who have ever practiced oral insertive sex, 10.0% were HPV-positive (n = 12), compared to 0.0% of those who have never practiced oral insertive sex specifically (n = 78) (Table 3). These data demonstrated a significant association between oral sex practice and HR-HPV status (p = 0.0057; phi coefficient = 0.21). Fourteen percent of those who have practiced oral sex in the last 6 months were HR-HPV positive (n = 57), compared to only 2.8% of those who have not practiced oral sex (n = 142). For the MSM that have performed oral receptive sex and oral insertive sex, oral/oropharyngeal HR-HPV prevalence was 7.5% and 10.0% respectively (Table 3). There was no association between insertive oral sex and any receptive oral sex.

**Table 3.** Association of sexual behaviour and HPV status.

Variable	Category	Overall		HPV status				p-value
				Negative		Positive		
		N = 199	%	N = 186	%	N = 12	%	
Current sex practice	Anal sex	138	69.4	*	*	*	*	
	Oral sex	57	28.6	49	86.0	8	14.0	0.0057
	Rimming	19	9.6	17	89.5	2	10.5	0.32
	Vaginal sex	1	0.5	*	*	*	*	
Number of partners in past 6 months	0	9	4.7	7	77.8	2	22.2	0.051
	1	53	27.7	52	98.1	1	1.9	
	2	39	20.4	35	89.7	4	10.3	
	3 or more	90	47.1	85	95.5	4	4.5	
	Unknown	8						
Sex practices ever done with a male partner	Oral receptive	161	80.9	149	92.5	12	7.5	0.13
	Oral insertive	121	60.8	108	90.0	12	10.0	0.0038
	Rimming bottom	92	46.2	86	93.5	6	6.5	0.80
	Rimming top	39	19.6	35	89.7	4	10.3	0.26
	Anal bottom	15	7.5	*	*	*	*	
	Anal top	5	2.5	*	*	*	*	

The results further indicate that more participants (46.2%) performed rimming bottom (having anus licked by partner) compared to rimming top (licking a partner's anus) (19.6%).

A significant association between rimming and HIV status (p = 0.0046; phi coefficient = 0.19) was observed. Of those who practiced rimming in the preceding 6 months, 94.7% were HIV-positive, compared to 63.9% HIV positivity in those who did not practice rimming (n = 180) during the same time (Table 4).

**Table 4.** Association with sexual behaviour and HIV status.

				HIV status				p-value
				Positive		Negative		
		Number	%	Number	%	Number	%	
				133	66.8	66	33.2	
Current Sex Practise	Oral sex	57	28.6	37	64.9	20	35.1	0.74
	Rimming	19	9.6	18	94.7	1	5.3	0.0046
Number of partners in past 6 months	0	9	4.7	5	55.6	4	44.4	0.58
	1	53	27.7	35	66.0	18	34.0	
	2	39	20.4	23	59.0	16	41.0	
	3 or more	90	47.1	63	70.0	27	30.0	

None of the study participants presented with any intra-oral HPV-associated or HPV-like lesions when examined. Other intra-oral abnormalities (non-HPV related) were observed in 26.1% (n = 52) of the participants and recorded as incidental findings. Within this group of 52 participants, the most commonly recorded oral/dental abnormality was dental caries (51.9%).

## 4. Discussion

Studies on HPV in MSM populations are generally limited to vaccination studies that focus on anal and/or penile infections as measurements of outcomes. Six percent of the 199 participants presented in this study had oral/oropharyngeal HR-HPV, and four of these were positive for HPV-31, and one for HPV-16. General reports on HPV detection in the mouth and oropharynx of South African populations and on oral/oropharyngeal tissue specimens are very limited and are reflected in Table 5. Oral/oropharyngeal HPV infection prevalence data amongst South African MSM are limited only to the Cape Town study, showing 11.5% oral HPV detection [6].

**Table 5.** South African studies reporting on oral/oropharyngeal HPV presence.

Authors	Year	Population sample	HPV types investigated
Wood et al. [27]	2020	149 Dental clinic attendees and 72 HIV-management clinic attendees	14 x HR, 3 x potential HR, 2 x LR; 12 x βHPV
Bulane et al. [28]	2020	449 OSCCa cases	16, 18, 31, 33, 45, 58
Sekee et al. [29]	2018	20 OSCCa cases	16, 18, 31, 33, 45, 58
Chikandiwa et al. [30]	2018	181 HIV seropositive men	37 HPV types
Muller et al. [6]	2016	200 MSM	24 x LR; 13 x HR
Davidson et al. [25]	2014	125 male factory workers	19xLR; 3pHR; 15xHR
Mbulawa et al. [21]	2014	221 heterosexual couples (442 participants)	37 HPV types
Vogt et al. [31]	2013	34 couples	15xHR; 22 other
Paquette et al. [32]	2013	55 OSCCa^†^ cases	37 HPV types
Marais et al. [33]	2008	115 women with confirmed cervical disease	37 HPV types
Richter et al. [34]	2008	30 women, oral scraping	19xLR; 3pHR; 15xHR
Boy et al., [35]	2006	59 OSCCa cases	16 and 18
Marais et al. [36]	2006	116 Dental clinic attendees	11, 16, 18
Van Rensburg et al. [37]	1996	146 OSCCa cases	6, 11, 16, 18
Van Rensburg et al. [38]	1995	66 OSCCa cases	6, 11, 16, 18

Note: * HR = High Risk; LR = Low Risk; pHR = probable High Risk; ^†^ OSCCa = Oral/Oropharyngeal Squamous Cell Carcinoma.

This study's prevalence of 6.1% falls within the lower end of the global prevalence range and is lower than the only other South African study, the Cape Town study focussed on MSM which was at 11.5% [6]. Although the HR-HPV prevalence in this study reflects lower than for the Cape Town study, the trend is the same. The global estimate of oral/oropharyngeal HPV prevalence in men is 5% and a wider spectrum of 3–57% is reported specifically for MSM.

Individual studies report a varying range of oral/oropharyngeal prevalence rates: In the UK King and colleagues [7] showed the prevalence of HR oral/oropharyngeal HPV in MSM was 5.9% comparable with the current report. Three other HPV oral MSM studies [8]–[10], report an oral/oropharyngeal HR-HPV prevalence of 2.0%, 9.0% and 17.0% respectively. Further in the spectrum, an Australian study reported one of the highest overall oral/oropharyngeal HPV-positivity in 37 of 170 rinses (21.7%) [11].

Data on the oral lesions or oral abnormalities found in the population described as MSM is sparse [2],[12], and head and neck HPV prevalence data for this group are limited to the extreme in South Africa [6]. As with other sexually transmitted pathogens, the most probable sources for acquiring oral/oropharyngeal HPV infection in an MSM population are the practice of oral sex (specifically fellatio), of rimming, and of using saliva as a lubricant for anal sex [13]–[15]. As a result of the perceived changes in sexual behaviour in addition to the higher prevalence rates of anal and penile HPV infection seen in MSM [2],[12], it is conceivable that the prevalence of oral/oropharyngeal HPV infection may be higher in this high-risk cohort than in the general population. Prevalence of oral/oropharyngeal HPV infection is estimated to be 4.5% in healthy individuals of all genders globally, but recent studies suggest that oral/oropharyngeal HPV prevalence among HIV-infected MSM may range between 20–45% [8].

This study found a much younger median participant age (27 years) than that of the Cape Town study (32 years) [6]. This suggests the probability that mainly younger individuals openly identify as MSM. Although no significant associations could be demonstrated, it is worth mentioning that education data revealed that 78.7% of MSM in this study completed high school and 47.1% completed tertiary studies. One hundred and five participants in this study were unemployed (53.3%) followed by 36.0% employed, and 10.7% students. The reported number of sexual partners had over the 6 years preceding this study was not associated with any of the parameters examined in this study population. In this report, HPV positivity is not significantly associated with age, race or with socio-economic status.

This report's MSM cohort mostly self-identified as black (98.5%). The Cape Town study [6] was conducted in an urban area in Cape Town, which could explain the racial demographic variation. It is difficult to correlate global statistics to sub-Saharan Africa as the majority of MSM recruited to global studies are Caucasian, and the MSM in this study were predominantly black participants. The catchment area for this study's participants is semi-urban and rural areas compromising mainly townships in which most of the residents are of African heritage.

In this study, 66.8% of the MSM participants were HIV seropositive and this group showed an HPV prevalence of 7.5% (n = 10) (Table 1). Oral/oropharyngeal HPV prevalence among HIV-infected MSM may range between 20–45%, which is considerably higher than the prevalence of MSM that are HIV negative [6],[8]. The three cities study in South Africa revealed HIV prevalence rates in MSM are 22.3% in Cape Town, 26.8% in Johannesburg, and 48.2% in Durban [16]. The finding that the majority of these MSM are HIV-positive (66.8%) is in line with other worldwide and local studies, albeit in the higher end of the spectrum.

Oral HPV has been reported with higher frequency in HIV-positive MSM greater than 40 years of age. A comparative study shows the majority of participants as Caucasian 96.5% with a median age of 39 years for HIV negative participants, and 44 years for HIV-positive participants [11]. The Cape Town study did not compare HPV presence within different HIV status groups, however, they found that 52.3% of their South African MSM participants were HIV positive, with 17.1% of these being oral/oropharyngeal HPV-positive (all genotypes) compared to 7.1% HPV presence in their HIV-negative participants.

An incidental finding from this study was that rimming was significantly associated with HIV-positivity. This could suggest that in this MSM population group, rimming is a risk factor to acquire HIV, and it is recommended that patients who identify as MSM, who have HPV infection, and report the practice of rimming, should be screened for HIV infection. The Cape Town study [6] interrogated the possible correlation between oral HPV incidence and HIV-positivity and suggested roles for HIV-related immune-suppression and risky sexual practises.

Despite the literature demonstrating an association between oral and oropharyngeal HPV infection and higher numbers of genital or oral sex partners [17]–[19], it was not the case for this study population. Differences in the same individual with regard to the number of oral sex partners and genital sex partners could partly influence any perceived lower oral HPV infection prevalence. Oral/oropharyngeal and genital HPV infections appear to be influenced differently when assessing these parameters in the contexts of gender and age associations but appear to have a higher prevalence in men than in women. The reasons for the reported increase in males are unclear despite investigations into co-factor exposure, gender differences in sexual behaviour, or even gender differences in natural disease progression [20]. Mbulawa and co-workers [21] confirmed that oral HPV infection in a South African population is acquired from sexual partners where the number of sexual partners and sexual practise increase the risk for oral HPV infection.

Infection by HPV is associated with an increased risk for HIV acquisition [22]. Reports suggest a higher HIV infection rate among MSM who are already infected with HPV [23],[24]. It is unknown whether HPV infection predisposes to subsequent HIV infection or is a marker of increased HIV infection risk. It is also unclear whether prevalent HPV infection or the immune response associated with clearing that HPV infection, or both, plays a role in potentiating HIV acquisition. The first evidence that found an association between HPV infection and increased risk of HIV acquisition came from studying MSM cohorts.

One meta-analysis showing a higher oral/oropharyngeal HPV prevalence in HIV-positive than in HIV-negative MSM also revealed a pooled prevalence for HPV 16 of 3.0% in HIV-negative MSM and 4.7% in HIV-positive MSM; a pooled prevalence of all HR-HPV types of 9.1% in HIV-negative MSM and 16.5% in HIV-positive MSM; and a pooled prevalence for the presence of any HPV type of 17.1% in HIV-negative and of 28.9% in HIV-positive MSM. The analysis confirmed that oral/oropharyngeal HPV presence was higher in HIV-positive MSM [12].

This study demonstrates that oral insertive sex is a significant risk factor in the acquisition of oral/oropharyngeal HPV infection in this population group (p = 0.0057). In contrast, Muller et al. [6] did not identify any independent risk factors associated with oropharyngeal HPV infection. Ninety-five-point five percent of the Cape Town study participants practised oral-receptive sex and 43.2% practised rimming [6]. This study found that a lower number of participants (80.9%) practised oral receptive sex when compared to the Cape Town study, while 60.8% performed oral insertive sex. This study revealed that oral/oropharyngeal HR-HPV presence was significantly increased in participants who performed oral insertive sex (10%), when compared to those who performed oral receptive sex (7.5%) (p = 0.0038) (Table 3). In our study, 14% of those MSM who reported to practice oral sex in the preceding 6 months were HPV positive.

In this study, HPV infection has a statistically significant presence in participants who performed oral insertive sex as opposed to those who performed oral receptive sex (p = 0.0038) (Table 3). The results further indicate that more participants (46.2%) performed rimming bottom compared to rimming top (19.6%) which is very similar to the figures reported by the Cape Town study [6]. In our MSM population a large number of participants (80.9%) practised oral receptive sex while 60.8% performed oral insertive sex. For the MSM participants who performed oral receptive sex and oral insertive sex, HR-oral HPV prevalence was 7.5% and 10.0%, respectively. The most commonly reported ‘current sex practice’ was anal sex (69.4%). The second most common sex practice was oral sex at 28.6% followed by rimming (9.6%). Significantly, 14% of participants who practised oral sex were positive for oral HR-HPV (p = 0.0057) compared to only 2.8% of those who did not practice oral sex. In the Cape Town study, 95.5% of the MSM practised oral-receptive sex and 43.2% practised rimming.

Data from Davidson et al. [25] shows that 40.8% of a South African male factory worker cohort (and only 3 of 7 HPV-positive participants) confirmed to practise oral sex. Two participants that were positive for HR-HPV types practised oral sex with more than one partner. Although oral sex was not practised by the majority of participants, it was practised by those who were positive with HR-HPV types. However, a recent study on oral sexual practices of dental clinic attendees from the general population in same catchment area determined that 18–35 years old tended to practice more oral sex, and 21.8% of the total cohort confirmed to practice oral sex [26]. Males reported a significantly higher prevalence of oral sex practice than the females, and at 32% compares favourably to that reported by Davidson et al. [25] and also of the current oral sex practice reported in this study of 28.6% (n = 57). Although sexual orientation was not interrogated by Wood and co-workers [26], another study by the group revealed that 64.7% of individuals started open-mouth kissing during their teenage years [27]. In the same population, 40.3% of participants gave oral sex to their partner, and 44.8% received oral sex from their partner. It is therefore conceivable that between oral sex practice and kissing, the spread of HPV could be higher. More studies into the different types of sexual practices in relation to oral and oropharyngeal HPV infection among South Africans are needed.

## 5. Conclusions

Data from South African, but specifically from a younger black MSM group, is very rare. The prevalence data points in this report cannot be extrapolated to the general South African MSM population but provides an important component to the baseline for research into oral/oropharyngeal HPV-infection of vulnerable MSM in South Africa and in Africa.

Although lower for MSM cohorts, the 6% HR-HPV oral/oropharyngeal prevalence in this study is in alignment with global reports. The prevalence of oral/oropharyngeal HPV in this MSM cohort was influenced by sexual practices. Different detection methods of the HPV, the difference in specimen collection methods, processing, storage, method of DNA extraction, sensitivity and specificity amongst the various global and local studies makes correlation challenging.

The significant associations found between oral insertive sex and oral/oropharyngeal HPV status (p = 0.0038; phi coefficient = 0.20), and between rimming and HIV status (p = 0.0046; phi coefficient = 0.19) supports the ano-oral-genital/genital-oral-anal HPV transmission routes in this population group, showing how HR-HPV gains access to the mouth and oropharynx, keeping in mind the HIV-seropositive rate of 66.8% in this group. Further studies can be designed to determine the natural history and burden of HPV-associated diseases in the South African MSM community, and also for the effective HPV prevention and public health strategies in these population groups. The current targeted interventions and HPV vaccination of boys to reduce the burden of HPV related diseases amongst MSM in South Africa requires further controlled prospective studies to provide the supporting evidence.

## Limitations

This lower HPV-positivity indicates the need for a much larger sample size and for a prospective approach which will be valuable to fully understand the dynamics of oral/oropharyngeal HPV infection in this local MSM population.

This population is almost exclusively black, and this is because of the geographic locality of the population. These data cannot be extrapolated based on race, and any race-based or ethnicity-based conclusions must be extremely carefully considered as it has immense potential for wrongful and inappropriate racial profiling. Even then, the authors strongly urge against this.

Although not quite a limitation, the lower HPV prevalence in this MSM which has a considerable HIV seropositive burden is curious and reinforces that further investigation into oral/oropharyngeal HPV transmission is needed in this population group.
